# Literacy predicts performance on a second-order false-belief task

**DOI:** 10.1371/journal.pone.0357313

**Published:** 2026-09-02

**Authors:** Tan Arda Gedik, Vania De la Garza, Ewa Dabrowska

**Affiliations:** 1 Department of Psychology, Bilkent University, Ankara, Turkey; 2 Lehrstuhl für Language and Cognition, Friedrich Alexander Universität Erlangen-Nürnberg, Erlangen, Germany; Educational Testing Service: ETS, UNITED STATES OF AMERICA

## Abstract

The vast majority of research on false-belief understanding has been conducted with literate populations. However, to our knowledge, no prior study has examined how literacy acquisition itself influences false-belief reasoning. The development of false-belief understanding is shaped by a range of factors, including language skills, education, reading experience, and cognitive abilities. Illiterate speakers often show substantial variability in language skills and cognitive abilities. In this study, we investigated the impact of nonverbal IQ, inhibitory control, and reading fluency on false-belief understanding in adult native Turkish speakers, comparing literate and illiterate participants. We found considerable individual differences in false-belief performance across both groups, as well as in the cognitive measures. Strikingly, reading fluency (assessed via a 1-minute timed word-reading task) emerged as the most significant predictor of false-belief performance, accounting for 49% of the variance. We propose that literacy acquisition may support the development of false-belief reasoning by enhancing language skills, cognitive control, metalinguistic awareness, and perspective-taking through engagement with written narratives. These findings carry broader implications for psychology, linguistics, and adult education.

## Introduction

Theory of Mind (ToM), the ability to understand others’ beliefs, desires, emotions, and intentions [1], is fundamental to successful communication and conflict resolution [2]. ToM develops throughout childhood, and many research studies have focused on this developmental period since adults are generally assumed to have fully developed basic ToM abilities. But emerging evidence suggests that even typically developing adults can experience difficulties in ToM reasoning under conditions of increased cognitive load or when they are required to suppress their own beliefs to accurately infer others’ mental states [3,4]. Importantly, deficits in ToM-related skills can lead to misunderstandings and communication breakdowns, highlighting the importance of understanding the factors that influence ToM across the lifespan [2].

One major milestone in many people’s lives is literacy acquisition, primarily through beginning formal education. Learning to read and write changes the wiring of the brain, improving the overall connectivity [5], and attending formal education improves nonverbal intelligence each year [6]. Given that education and cognitive skills play key roles in the development of false-belief understanding, a core, basic component of ToM, studying ToM in populations that have had limited literacy experience (i.e., illiterate adults) is essential not only to broaden the empirical scope of cognitive science but also to shed light on how literacy and education influence adult ToM reasoning.

One of the most common tools for measuring ToM, especially in developmental studies, is the false-belief (FB) task. FB tasks assess the ability to recognize that others can hold beliefs that differ from one’s own knowledge. FB tasks vary in complexity. First-order FB tasks assess whether an individual understands that someone else can hold an incorrect belief (e.g., a child understanding that Sally will look for her ball where she left it, despite knowing it has been moved). Second-order FB tasks require reasoning about what one person believes about another’s belief (e.g., understanding that John thinks Mary believes the ice cream is still in the fridge, although Mary saw it moved). While children typically pass first-order FB tasks by ages 4–5, mastery of second-order FB reasoning generally emerges between ages 8 and 10 in literate segments of the population [7,8]. False-belief understanding is rarely tested with typically developing adults “because nobody really doubts that a typical adult has such basic mind reading concepts” as stated by Apperly on page 74 [9] and most ToM tasks for adults (including false-belief tasks) show ceiling effects and are not suitable for analyzing individual differences in false-belief understanding [10]. Nevertheless, FB tasks offer a valuable, low-cognitive-load method to assess ToM in adults, especially under conditions where cognitive demands may affect performance in ToM related tasks [4].

Despite the claim that ToM remains stable and constant throughout adulthood in the absence of cognitive deficits [11], recent research has demonstrated that ToM in adulthood is influenced by various external factors [12–14]. As discussed in [14], individual differences in adult ToM are largely driven by cognitive changes across the lifespan, though the impact of these changes depends on other cognitive and experiential factors. These factors include alterations in brain regions involved in ToM reasoning [15,16], fluid intelligence [17], social experiences [18], inhibitory control [13], bilingualism [14], and language ability and reading practices [12].

### Language, reading, and ToM

Among the factors mentioned above, language ability (in this study, we refer to linguistic abilities as strictly lexis and grammar), particularly syntactic and vocabulary knowledge, plays a crucial role in facilitating early successful expression and comprehension of mental states [19]. However, there has been disagreement regarding the significance and strength of this relationship [20]. According to some researchers [21–24], this relationship may exist either because FB tasks are inherently verbal, or because the cognitive processes involved in FB reasoning overlap with those required for language processing. In contrast, other researchers maintain that language plays a causal role in the development of FB understanding [25,26].

Given that language may play a role in FB understanding, one could expect a relationship between FB understanding and literacy for at least two reasons. First, having a larger vocabulary [19] and mastering certain linguistic structures, such as embedded clauses [27,28], enhances performance on FB tasks. These structures and lexical items appear more frequently in written language [29], and adults who read more tend to perform better on linguistic tasks tapping into grammar and vocabulary [30–32]. Therefore, reading may contribute to improved FB understanding by enhancing overall language proficiency in the first language.

The second reason concerns the Matthew Effect in reading and cognitive development [33]. This effect describes a self-reinforcing cycle in which early advantages in reading lead to cumulative gains in linguistic and cognitive skills, while limited exposure results in individuals falling further and further behind over time. In the context of FB understanding, extensive reading experience can strengthen cognitive functions such as inhibition and working memory, both essential for accurately representing others’ mental states. Furthermore, reading often involves engaging with narratives that require the reader to infer characters’ beliefs, intentions, and emotions, effectively simulating social experience. Thus, throughout both childhood and adulthood, regular reading may support the development and refinement of FB reasoning by continuously exercising the cognitive and social processes it depends on.

Over the past fifteen years, research on the impact of reading narrative fiction on empathy and ToM has grown significantly (for reviews, see [34]). Most studies involving adults in this field use the Reading the Mind in the Eyes test or the Interpersonal Reactivity Index to assess ToM or empathy, while reading habits are typically measured using the Author Recognition Test (ART), which asks participants to identify authors from a list containing both real and fictitious names. Several studies have linked lifetime exposure to narrative fiction with enhanced ToM and empathy (see [35] for a meta-analysis). In contrast, exposure to expository or non-narrative texts shows no such benefit, and is sometimes even negatively associated with sociomoral outcomes.

Experimental studies have also investigated whether brief exposure to narrative fiction can influence performance on ToM tasks. Although findings are mixed, a recent meta-analysis [12] reports a small but positive effect of fiction reading on ToM skills. They further suggest that individuals who are already socially skilled may be more inclined to read fiction, possibly because of its rich social content (see also [36]). Supporting this, [37] found a small positive relationship between fiction reading and FB understanding in older adults. While these findings suggest that reading fiction contributes to ToM, the mechanisms behind this effect currently remain unclear.

One possibility is that the ToM benefits of reading stem not from exposure to narrative fiction per se, but from frequent engagement with linguistic environments rich in mental state language. Fiction may simply be one of many sources, albeit a particularly potent one, of such input. In that case, the observed links between reading and ToM might reflect a broader relationship between literacy and exposure to abstract, recursive, and socially complex language, which is prevalent in written forms of language.

In light of the foregoing discussion, understanding the role of reading in ToM development is especially important in global contexts where literacy is not universal. In different societies, some segments of the population remain illiterate due to structural inequalities. In Turkey, for example, an estimated 4.9% of the female population over age 15 is illiterate [38], reflecting a broader global trend [39]. Therefore, the foregoing discussion invites a crucial question: How is FB understanding influenced in the absence of literacy skills?

### Literacy, other cognitive abilities, and ToM

Given the influence of cognitive and experiential factors on ToM in adulthood, literacy and educational experiences warrant specific consideration due to their broad cognitive impact. Prior studies [40,41] have found significant differences between literate and illiterate adults in nonverbal IQ; however, executive function has yet to be thoroughly investigated in illiterate populations. Additionally, literacy and schooling enhance working memory [42], a domain strongly implicated in ToM performance [43]. Literacy has also been shown to support the acquisition of complex syntactic structures, with recent research suggesting that illiterate speakers may have limited mastery of complement clauses, relative clauses, passives, and nominalizations [40,41,44].

Illiterate adults typically lack access to formal schooling, which cultivates crucial social-cognitive skills such as perspective-taking, debate, and empathy, all known to support ToM development [20]. Educational experience also shapes core cognitive domains including nonverbal IQ [6] and executive functioning [45], both integral to ToM reasoning [46]. It has been shown that each additional year of schooling is associated with an increase of 1–5 points in nonverbal IQ [6], which helps explain why illiterate adults tend to score lower on such tasks [40,41]. Other studies have found that higher nonverbal IQ is consistently linked to better performance on ToM tasks [24,46–48]. For instance, Apperly and colleagues [47] found that nonverbal IQ significantly predicted success on complex ToM tasks across both children and adults. Supporting this behavioral evidence, neuroimaging studies reveal that both nonverbal reasoning and ToM tasks recruit overlapping brain areas, particularly in the prefrontal cortex, suggesting a shared neural substrate [49,50].

There is also some evidence suggesting that executive functions – top-down control processes like working memory, cognitive flexibility, and inhibitory control – are important for FB understanding [51]. Inhibitory control skills are particularly relevant in this context, since individuals must inhibit their own knowledge of reality to represent another person’s mistaken belief. In children, the relationship between inhibitory control and FB understanding remains significant even when age and language abilities are accounted for [52,53]. In adults, however, the evidence is more mixed, with some studies finding strong associations and others reporting weak or no effect at all [13,54]. One possible explanation lies in the neural overlap between inhibition and ToM: both rely heavily on prefrontal regions of the brain, which continue to develop well into adulthood [15,55,56]. Additionally, both may draw on similar cognitive resources such as working memory, and attentional control [57,58]. Notably, several studies suggest that literacy and formal education support the development of executive functions [45,59,60], raising the possibility that reduced inhibitory control may underlie some of the difficulties illiterate adults face in ToM tasks.

Finally, literacy is often correlated with socioeconomic status (SES) (e.g., [61]), which itself can impact FB understanding, particularly in children [62]. This may create a cycle in which children from illiterate households or with illiterate caregivers have fewer opportunities to engage with cognitively enriching environments, such as libraries, museums, or theaters. Moreover, illiteracy tends to be concentrated in peripheral urban neighborhoods or rural areas, where logistical barriers further restrict access to these resources. The influence of SES on development is also well documented in language acquisition research, which shows that children from lower SES families often lag behind their peers in linguistic success (e.g., [63]). Taken together, these factors suggest that illiterate individuals may be at a disadvantage in FB understanding.

### Current study

Given the importance of preserving healthy ToM abilities across the lifespan for well-being, successful aging, and interpersonal functioning [64], it is crucial to investigate how literacy, as an external factor, influences ToM, either directly or indirectly through domain-general cognitive abilities. The present study seeks to address this gap by examining FB understanding in literate and illiterate adult native Turkish speakers using a standardized FB task. Specifically, we aim to determine whether illiterate and literate adults differ in their FB understanding performance, and to what extent any observed differences can be explained by variation in nonverbal IQ, inhibitory control, literacy, or a combination thereof.

Our primary research questions are: (1) Do literate and illiterate speakers differ significantly on FB tasks? (2) Are these differences attributable to literacy itself or other variables confounded with it (especially inhibitory control and nonverbal IQ)? To answer these questions, we tested literate and illiterate Turkish speakers. The choice of this population was entirely based on availability. Performance on FB tasks serves as the dependent variable, with reading ability, inhibitory control, and IQ as key predictors, and age included as a covariate.

## Methods

### Participants

We collected data from 41 female illiterate (mean age = 48.58) and 35 female literate native Turkish speakers (mean age = 45.51). Illiterate speakers were based in Ankara, Turkey and at the time of the study had attended the adult education center for the literacy course for a short period of time (mean = 6.09 months, SD = 7.49). During this course, they receive literacy education alongside subjects such as math, basic history, and Turkish for 80 teaching units, and each teaching unit is 40 minutes. When participants successfully finish the course, they receive a degree that is equivalent to the primary school diploma in Turkey. Literacy centers screened illiterate speakers for various cognitive impairments and speech disorders, and participants with such diagnoses were excluded from the study.

While many of our participants were fully illiterate (i.e., could not read even simple words), others were able to read and write very basic Turkish (i.e., their names, phone numbers, short sentences and short picture books for very young children). As shown in the results section, illiterate participants on average could read 9 words, with 4 outlying participants reading just as much if not more words than literate speakers. On the other hand, literate speakers could read about 10 times more words per minute. However, these abilities were newly acquired. In other words, unlike the literate controls, illiterate speakers did not have a lifetime of reading experience.

Our literate speakers learned to read and write at around the age of 6–7, and had received formal education (mean = 17.34 years, SD = 4.38). They were also based in Ankara, Turkey. All literate speakers had finished a high school degree at the very least. 13 participants had a BA degree, 9 received an MA degree, 3 received a PhD degree, 6 had a pre-BA education, and 4 had a high school education. This study was approved by the Bilkent Ethics Committee (2022_12_21_01). We obtained written consent from literate participants, and oral consent from illiterate participants upon reading to them the consent form. The recruitment period for this study was from 1-March-2024 to 1-June-2024.

### Materials

#### Second order false-belief tasks.

**Fig 1:**
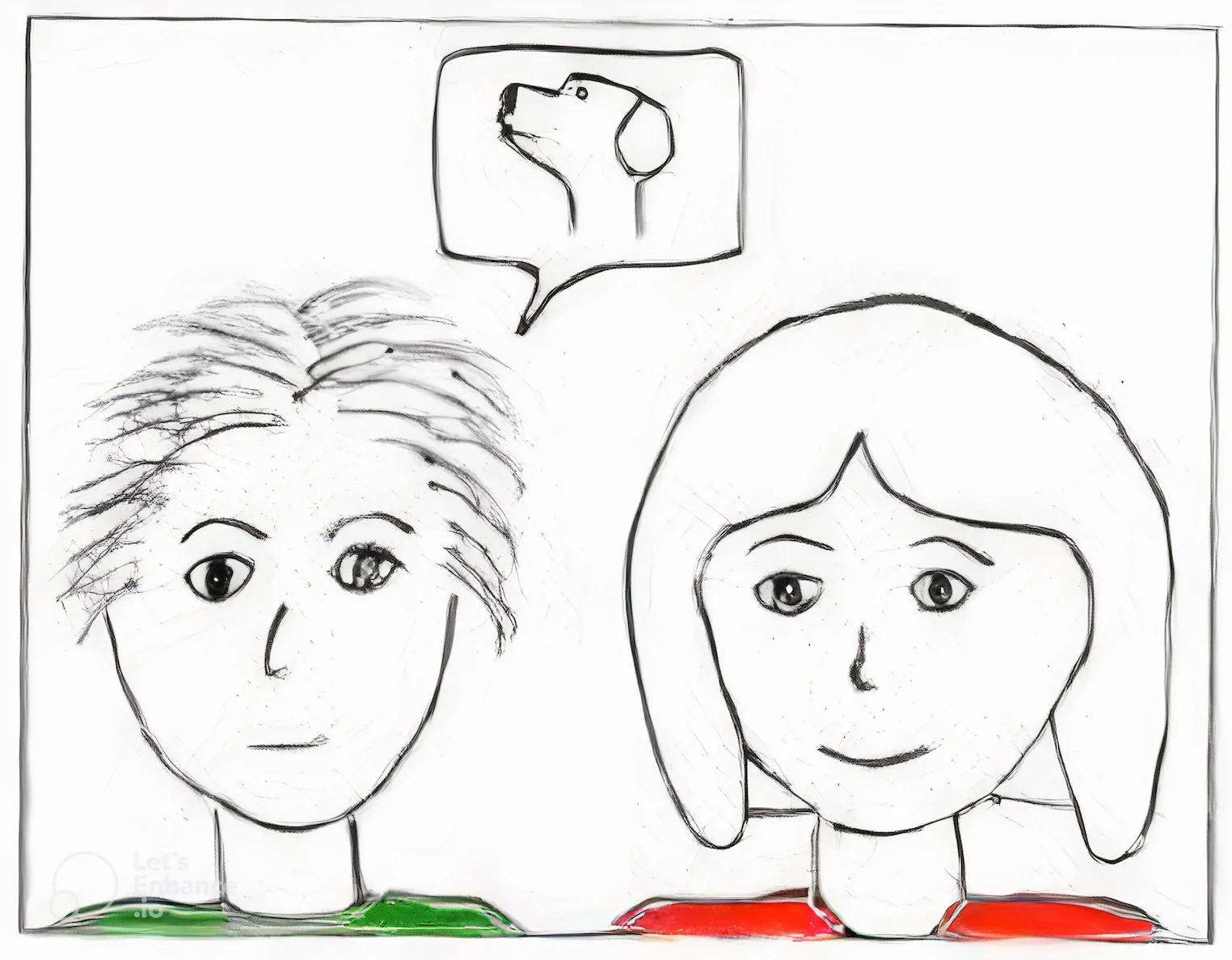
The first picture used in conjunction with the puppy story.

We used a second order false-belief task to test FB understanding (see S5 File for the link to the stories) and administered it orally, that is the experimenters read the stories to the participants in a neutral tone and a slow pace. FB tasks require participants to attribute a false-belief to another person and predict their following actions based on that belief [65]. This task consisted of two stories in which the participants needed to infer a character’s belief about another person’s belief. Throughout both stories, we used the drawings created by Liesbeth Flobbe (see S2 File for images). These were enhanced using AI (letsenhance.io, see Fig 1), since the original drawings were poor in quality and could have been difficult to decipher for participants with little experience with drawings. The two stories, the chocolate bar story [66], and the birthday present story (i.e., referred to as puppy story) [67] were designed to measure children’s FB understanding and were adapted to Turkish for use with Turkish children by [68]. Both stories have redundant parts such that the key information to succeed in the FB questions is repeated twice in both stories (see the stories in the file provided above). Both stories use basic vocabulary and relatively simple syntax which should be understood by children as young as 4 (see Table 1 in S4 File for a frequency analysis of the words.

**Table 1:** Mean scores as percent correct (standard deviation in parentheses) on subcomponents within each FB task and overall, both FB traditional and Sensitive FB.

		RQ	1^st^ Order	LC	2^nd^ Order	Justification	Traditional FB score	Sensitive FB
***Illiterate***	Puppy	43% (50%)	41% (62%)	80% (40%)	31% (47%)	19% (40%)	7% (26%)	30.6% (26%)
	Chocolate	48% (50%)	53% (50%)	95% (21%)	70% (46%)	17% (38%)	34% (48%)	47% (26.6%)
	Total	46% (50%)	47% (50%)	87% (32%)	51% (50%)	18% (38%)	20.5% (31.5%)	39% (19.8%)
***Literate***	Puppy	94% (23%)	62% (49%)	94% (23%)	71% (45%)	68% (47%)	68% (47%)	67.3% (32.6%)
	Chocolate	97% (16%)	91% (28%)	100% (0%)	97% (16%)	88% (32%)	94% (23%)	92.3% (19.6%)
	Total	95% (20%)	77% (42%)	97% (16%)	84% (36%)	78% (41%)	81% (27%)	80% (21.6%)

*Notes.* RQ= reality question, LC= linguistic control.

In the first story (the birthday present story), a mother wants to surprise her son with a puppy but tells him that she instead bought him a toy. At this point, the experimenter asks the first reality control question (“what did the mother get her son as a gift?”). The son enters the basement to get his roller-skates, where he finds the puppy. At this point, the experimenter asks the 1^st^ order ignorance question (“does the boy know that the mother got him a puppy as a gift?”), and the linguistic control question (“does the mother know that the son saw the puppy?”). Then, the grandmother calls the mother to ask about the birthday celebration and what the boy thinks he will receive as a gift. The experimenter asks the 2^nd^ order belief question at this point (“What will the mother say to the grandma?”), followed by a justification question (“why does she think that way?”).

In the second story (the chocolate bar story), a boy places a chocolate bar in a drawer, and while he is in a different room, the sister moves the bar to a toy-box. The boy witnesses this through a window, although the sister is not aware of this. The experimenter asks the reality control question at this point (“where is the chocolate bar now?”), then the 1^st^ order ignorance question (“does the boy know that the sister moved the chocolate bar to the toy box?), followed by the linguistic control question (“does the sister know that the boy sees her relocating the bar?), and then the 2^nd^ order FB question (“where does the sister think that the boy will look for the chocolate?). This is followed by a justification question (why does she think that way?).

We used two scoring methods: one that uses the traditional method to keep compatibility with previous studies and a more sensitive one that tests a wider range of FB skills. In the traditional method, participants were awarded 1 point per story if they answered both control questions and the 2^nd^ order FB question correctly, and the maximum score in the traditional method is 2 (1 per story). In the sensitive method, per story, participants were awarded 1 point if they answered the reality control question and 1^st^ order ignorance question, another point if they answered both control questions and the 2^nd^ order FB question, and another point if they answered the justification question correctly; the maximum score in the sensitive method is 6 (3 per story). The inter-rater reliability of two native Turkish speakers for the raters was found to be *k* = 0.87 (95% CI: 0.79–0.95), indicating substantial agreement.

We used a second-order FB task for two reasons. First, second-order FB tasks are relatively difficult: previous studies have shown that children’s performance on such tasks continues to develop throughout childhood and adolescence, and even adult participants do not always perform at ceiling [8]; therefore, we can expect a considerable amount of variation. [66] demonstrated the robustness of these tasks in gauging advanced ToM abilities, making them suitable for distinguishing between varying levels of cognitive development among adults. Second, the task used in this study involves stories about familiar situations and is thus relatively non-threatening. Last but not least, the task has been administered to Turkish speaking children and adults [8], thus providing a point of comparison for our results.

#### Nonverbal IQ measure: Raven’s CPM.

Illiterate adults tend to obtain lower scores on nonverbal intelligence measures compared to literate adults. Raven’s Colored Progressive Matrices (CPM) has often been used in studies involving illiterate populations (see, for example [40,41]), giving us a point of reference for comparison. It consists of three sets, each containing 12 problems of increasing complexity. Each problem presents a series of patterns with a missing component. The task for the respondent is to identify the missing part from a selection of options provided at the bottom of the page. A point is granted for each correct answer, allowing for a maximum score of 36.

#### Inhibitory control task: Emotional stroop task.

We used the Emotional Stroop Task [69] to measure inhibitory control. In this task, the participants see a happy or a sad face emoji and they are instructed to say the opposite of the stimulus: when shown a happy face, they need to say sad and vice versa. We used emojis as our stimuli because based on our initial meetings with the illiterate participants; they all had smartphones and used WhatsApp and other social media platforms where emojis were present. There are two reasons behind choosing this task over other executive function tasks: first, traditional inhibitory control tasks use written language, and we needed to accommodate our illiterate participants. Second, this task has been demonstrated to be a good measure of inhibitory control and can capture individual differences [69].

Before administering the task, the experimenter introduced the task as a “say the opposite” game. First, there was a trial phase with 4 random happy and sad face stimuli. In the trial phase, participants received feedback. After the trial phase, they saw 5 happy and 5 sad faces in random order, and no positive or negative feedback was provided. If there were 4 errors in a row, the experimenter reminded the participant of the rule of the game. Each correct response counted as 1 point, and a participant could score between 0–10 given that there were 10 trials.

#### 1-Minute word reading task.

We used the Turkish 1-Minute Word Reading Task, developed by [41] to cater to both literate and illiterate speakers, as conventional literacy measures, such as author recognition tests, would not be applicable for illiterate individuals. Similar tasks have been created for English and Greek languages as well [70–72]. Previous research indicates a modest yet statistically significant correlation between scores achieved on such tasks and adult vocabulary size [71], along with a moderately substantial correlation between words read per minute and text comprehension in children [73].

The task comprises 120 real words and 120 pseudo words (i.e., words that look and sound like Turkish but do not exist). In each condition, the words increase in length, with four different letter conditions for both real and pseudo words (for real words: 5, 6, 7, 8 letters; for pseudo words: 6, 7, 8, 9 letters). Each segment (real and pseudo word) is timed for one minute. Participants are initially provided with 20 extra words for practice reading aloud accurately. Subsequently, they are instructed to read as many words correctly as possible within one minute, separately for real and pseudo words. Each correctly read word earns one point, while each misread word incurs a penalty of −1 point. If a participant corrects a misreading, they regain the lost point. We created a composite score of real and pseudo word reading by converting each into z-scores and computing the average of the two (see also the R script).

### Data analysis

We imported the data into R (version 2023, 4.3.1) for statistical analyses. Using standard linear regression modeling, we examined the effects of inhibitory control (IC), IQ, age, and reading fluency (1-minute word reading task) on false-belief (FB) understanding, scored with the sensitive method. All four predictors were initially entered into the model. Because reading fluency and group were very strongly correlated (r = .94, p < .001), we considered whether to include reading fluency or group as a predictor. We selected reading fluency, as its continuous nature offers greater sensitivity and serves a comparable function to the group variable.

Model selection followed Crawley’s [74] recommendation: non-significant predictors were sequentially removed based on log-likelihood tests, and predictors that did not improve model fit were excluded. This process was repeated until all remaining predictors were significant. To aid interpretation, all variables were mean-centered using the scale function in R. Group comparisons were additionally evaluated with independent-samples t-tests using the t.test function and the lsr package [75], which also provided effect sizes. Assumptions of linear regression, including checks for multicollinearity, heteroscedasticity, autocorrelation, and outliers, were tested using the performance package [76], and all models met these assumptions.

### Procedure

We tested our illiterate participants in a quiet familiar room at adult education centers, and literate speakers in a quiet office room at a university building. Literate speakers read and signed the consent form, whereas the experimenters read the consent form to illiterate speakers and received their formal consent. We first collected demographic data: age, how long our participants have been learning literacy (for literate speakers this was how long they have been literate), how long they attended formal schooling, their highest attained degree, and if they received formal literacy instruction prior to the adult education center they were attending. The 1-Minute word reading task was administered, followed by the inhibitory function task, then the nonverbal IQ measure, and the two theory of mind tasks. The theory of mind tasks were administered orally by the experimenters, and the stories were read in a neutral tone with a slow pace one by one. Except for the 1-minute word reading task and the nonverbal IQ measure, all the materials were presented on a computer screen operated by the experimenter. Each session took around 15–20 minutes. During the experiment, the experimenter consistently reiterated the prompt with a neutral intonation whenever participants hesitated or requested to hear it again. Additionally, the experimenter recorded participants’ responses on a separate computer. We prioritized the comfort and well-being of illiterate speakers during the study. The experimenter consistently offered positive reinforcement, such as “Yes, good” and “You’re doing very well,” and regularly checked in to ensure participants’ comfort and willingness to continue after each task. See also the questionnaire on inclusivity on global research filled out by the authors (see S3 File).

Materials, data, and the code are available at the following link:  https://osf.io/9mhgv/overview?view_only=ce541519b9da4f0983a7d9beead4b09c

## Results

Table 1 presents the scores as percent correct (standard deviation in parentheses) for individual components of the FB task. As described in the methods section, two scoring methods were used: a traditional method to ensure comparability with prior studies, and a more sensitive method. In the traditional method, participants received a maximum score of 2, with 1 point awarded per story if they answered both control questions and the second-order false-belief (FB) question correctly. For the sensitive scoring method, please see above.

Across both groups, overall and condition-specific scores indicate that the chocolate story was easier than the puppy story. Literate participants outperformed their illiterate counterparts across all components, though individual variation was evident, as shown by the standard deviations. Three specific observations are noteworthy for the illiterate group. First, their near-ceiling performance on the LC suggests that they comprehended the core narrative structure of the task, and the second-order FB question essentially requires the same information. Second, despite the stories being supported by visual aids, the illiterate participants did not reach ceiling-level performance on the RQ, which is somewhat surprising. Third, they exhibited difficulty with the first-order ignorance question and, interestingly, performed slightly worse on it than on the second-order FB question. This pattern may be partly explained by the scoring scheme: to receive 1 point on the first-order FB question, participants needed to answer the RQ correctly, whereas the second-order score required correct responses to both the RQ and LC.

The data in Table 2 highlights significant differences between illiterate and literate groups across various cognitive and educational variables. As can be observed, the most striking differences are the number of years spent in formal education, nonverbal IQ, executive function, and FB understanding. On average, illiterate speakers attended roughly a little less than half a year of formal schooling without the literacy course, whereas literate speakers are educated at about 17 years on average.

**Table 2:** Descriptive statistics for the variables per group, standard deviation in parentheses, t-tests and effect sizes (Cohen’s d).

	Illiterate		Literate		Comparison illiterate vs literate			
	Mean(SD)	Range	Mean(SD)	Range	df	t-value	p-value	Cohen’s d
**Age**	48.58(11.02)	21-65	45.51(11.16)	28-68	71.85	1.23	p = .23	0.28
**Education**	0.65(1.38)	0-5	17.34(4.38)	11-27	39.82	−21.59	p < .001***	5.30
**IQ**	11.83(4.72)	4-21	30.46(4.62)	18-35	72.58	−17.31	p < .001***	3.97
**IC**	6.02(3.24)	0-10	9.11(1.98)	4-10	67.45	−5.08	p < .001***	1.12
**FB traditional**	0.41(0.63)	0-2	1.62(0.54)	0-2	73.98	−8.89	p < .001***	2.04
**FB sensitive**	1.09(1.41)	0-6	4.54(1.46)	2-6	71.26	−10.40	p < .001***	2.40
**Reading fluency**	−0.85(0.24)	−1.00–0.08	1.01(0.38)	0.12-1.74	56.13	−254.99	p < .001***	5.94
**Read Real**	9.73(16.24)	0-73	99.06(17.15)	56-120	70.74	−23.18	p < .001***	5.35
**Read Pseudo**	2.56(4.73)	0-20	60.31(15.98)	22-93	39.10	−20.61	p < .001***	5.07

*Notes:* Education = average months spent in formal schooling, IQ = nonverbal IQ (Max = 36), IC = inhibitory control (Max = 10), FB traditional = false-belief traditional scoring (Max = 2), FB sensitive = false-belief sensitive scoring (Max = 6), Read Real = real words in the word reading task (Max = 120), Read Nonce = pseudo words in the word reading task (Max = 120), p < 0.001 ‘***’

The nonverbal IQ test showed high reliability in our sample (split-half reliability = .95), comparable to previous findings in Turkish-speaking population (.80) [77]. Our illiterate participants had a mean nonverbal IQ score of 11.83, which may initially appear low. However, norming data [77] place these scores within the range observed for younger children (25^th^ percentile for children 3;9–4;2), and comparable results have been reported for minimally educated adults. For example, [78] found a mean CPM score of 13 among adults with 0–4 years of education, slightly above the performance of our participants, who averaged only 6.5 months spent in formal schooling. A recent meta-analysis [6] further demonstrates that each additional year of education is associated with a 1–5 point increase in IQ scores. Together, these findings suggest that our participants’ performance reflects limited formal schooling rather than cognitive impairment.

**Fig 2:**
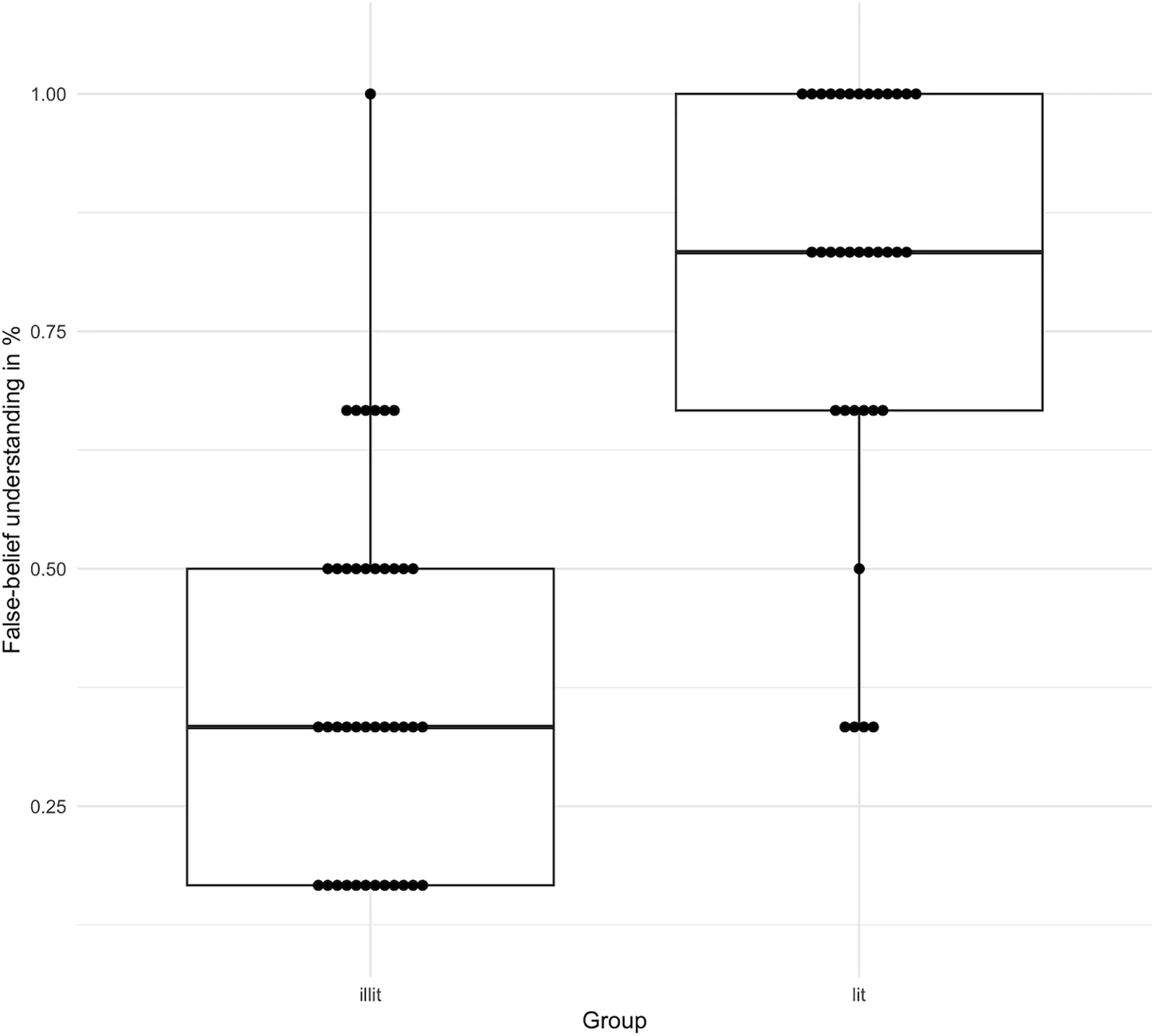
FB Scores (sensitive scoring) with individual performance (percentage) per group.

Fig 2 shows individual and group performance on the FB tasks. As can be clearly observed, there is some overlap between the two groups. Interestingly, there is only one illiterate participant who is at ceiling, whereas a large proportion of the literate speakers are at ceiling. Also of interest is the four literate participants that were within the performance range of illiterate speakers.

**Fig 3:**
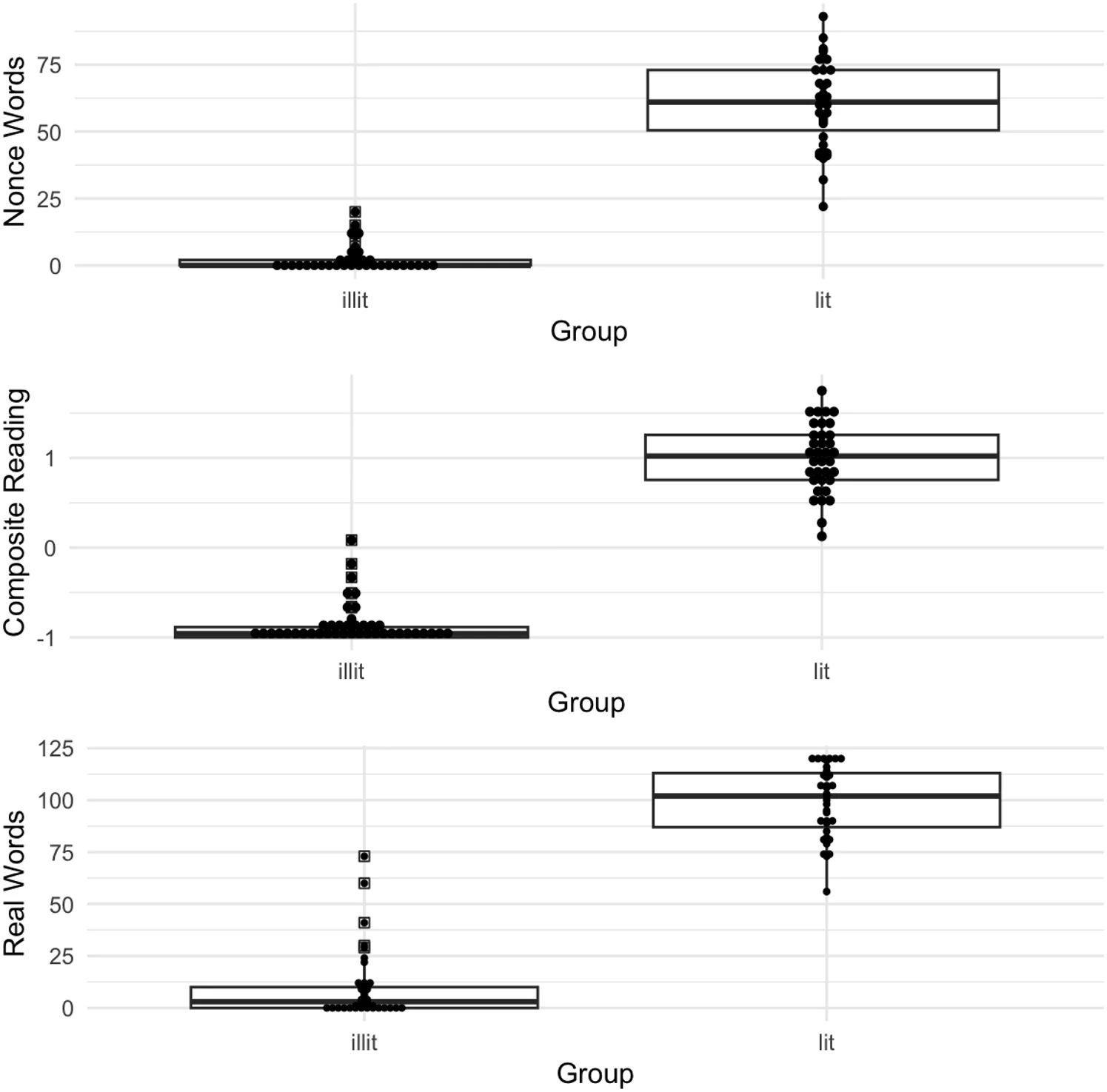
Reading measures per group. *Note*. Composite reading = Composite reading score of real and pseudo words, Read Real = real words, Read Nonce = pseudo words.

**Fig 4:**
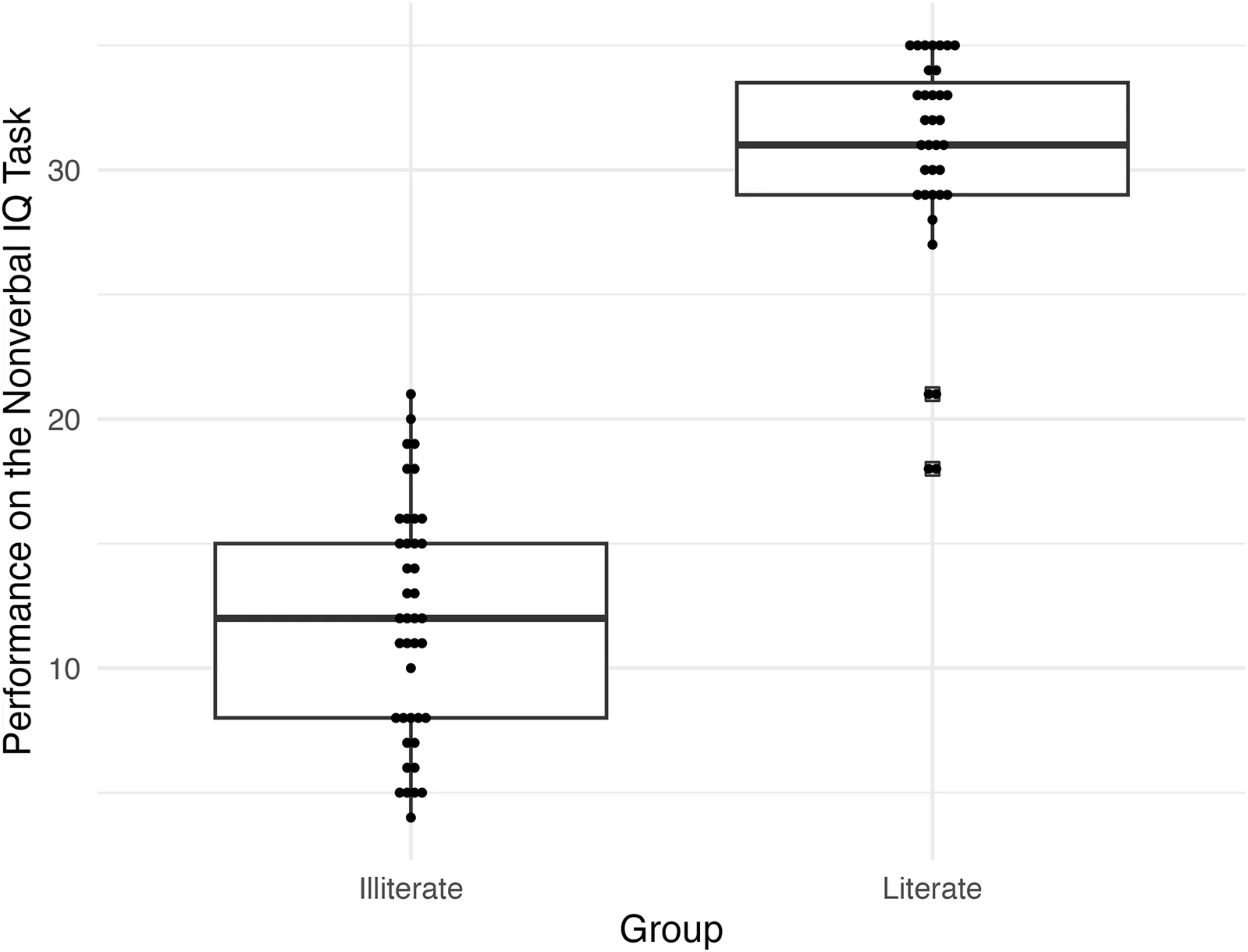
Raw IQ scores in the two groups.

**Fig 5:**
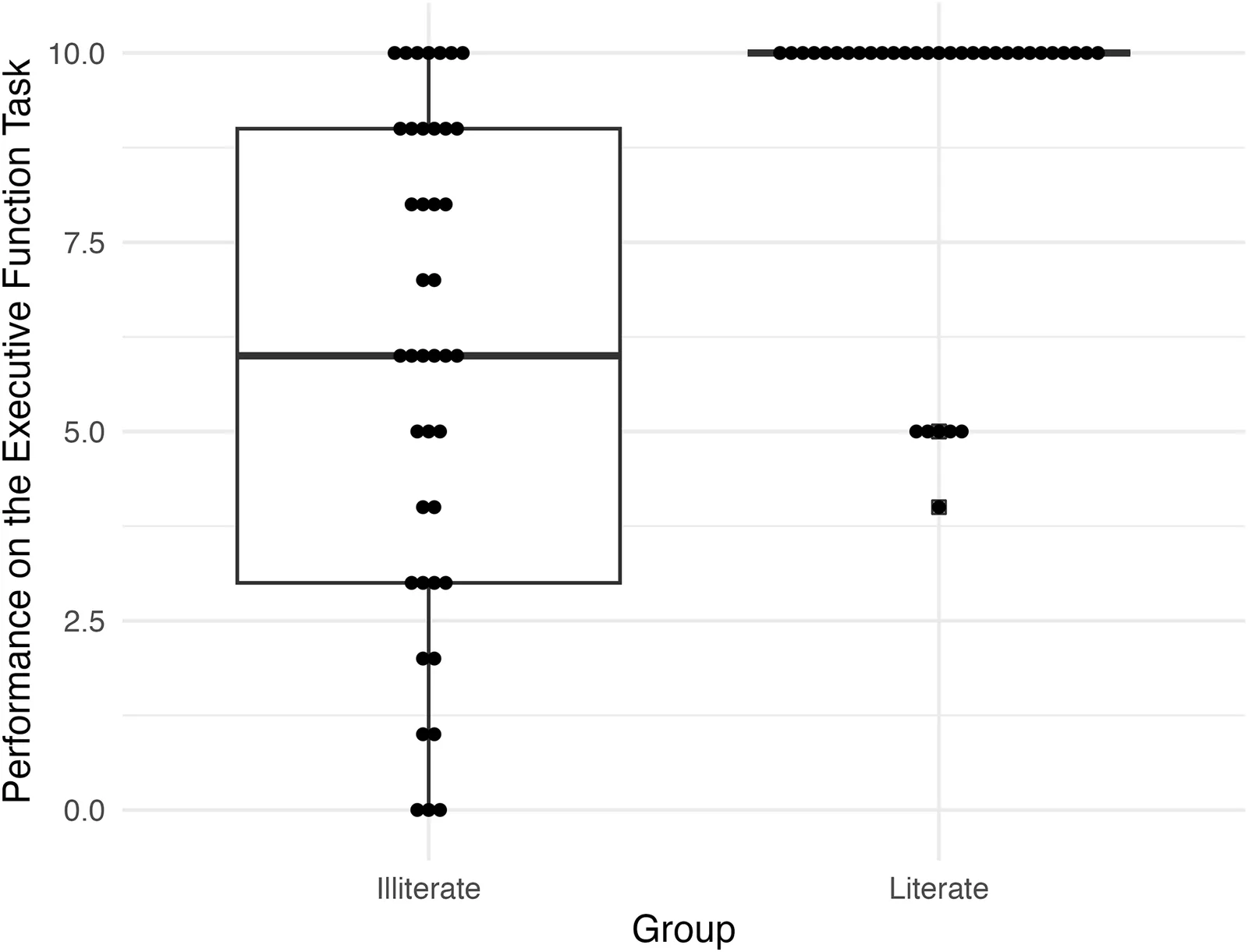
Performance on the executive function (inhibitory control) task per group.

Figs 3–5 show individual and group performance on reading, IQ, and IC, respectively. Unsurprisingly, there is virtually no overlap between the two groups’ performance on reading (Fig 3). The performance on nonverbal IQ (Fig 4) shows a large difference between the two groups, but also considerable individual differences within groups. Finally, and perhaps most strikingly, in the IC task (Fig 5), there are again large individual differences. While the original creators of the IC task do not report a split-half reliability score (but only a test-retest score, [69]), our analyses show that the task was highly reliable with a split-half reliability of  .90. Scores ranged from 0 to 10, as there were 10 trials.

To investigate the effects of our variables on FB understanding, we fitted a linear regression model (see section 2 for details). All variables were scaled and figures reported under the column estimate in Table 3 and other regression models in the supporting files are standard coefficients. Table 3 presents the results of the final best fitting regression model for FB scores. As is clear from the table, composite reading displays statistical significance at p < .001, and it accounts for a remarkable 49% of the variance in FB abilities. Age is also significant but only accounts for about 6% of the variance. Comparing the LMG metrics in Table 3 and in S4 Table, it appears that reading fluency does the majority of the explanation, and the other predictors are superfluous. Finally, education and reading exhibit a strong positive correlation across the entire dataset at  .92, but not within specific subgroups (see S1 Table, S2 Table, and S3 Table). Due to multicollinearity, it was not feasible to include both variables in the same statistical model. However, we conducted an analysis using education as the primary variable instead of reading fluency (see S5 Table), and the results were highly comparable with those obtained when reading was used.

**Table 3:** Final best fitting model (predicting sensitive FB).

Predictor Variables	Estimate	Standard error	*t* value	Pr(> |t|)	lmg
**Intercept**	0.00	0.07	0.00	1.00	
**Age**	−0.18	0.07	−2.29	0.02	.0639
**Reading fluency**	0.68	0.07	8.65	0.000	.4899
**Adjusted R²**					.5416

**Fig 6:**
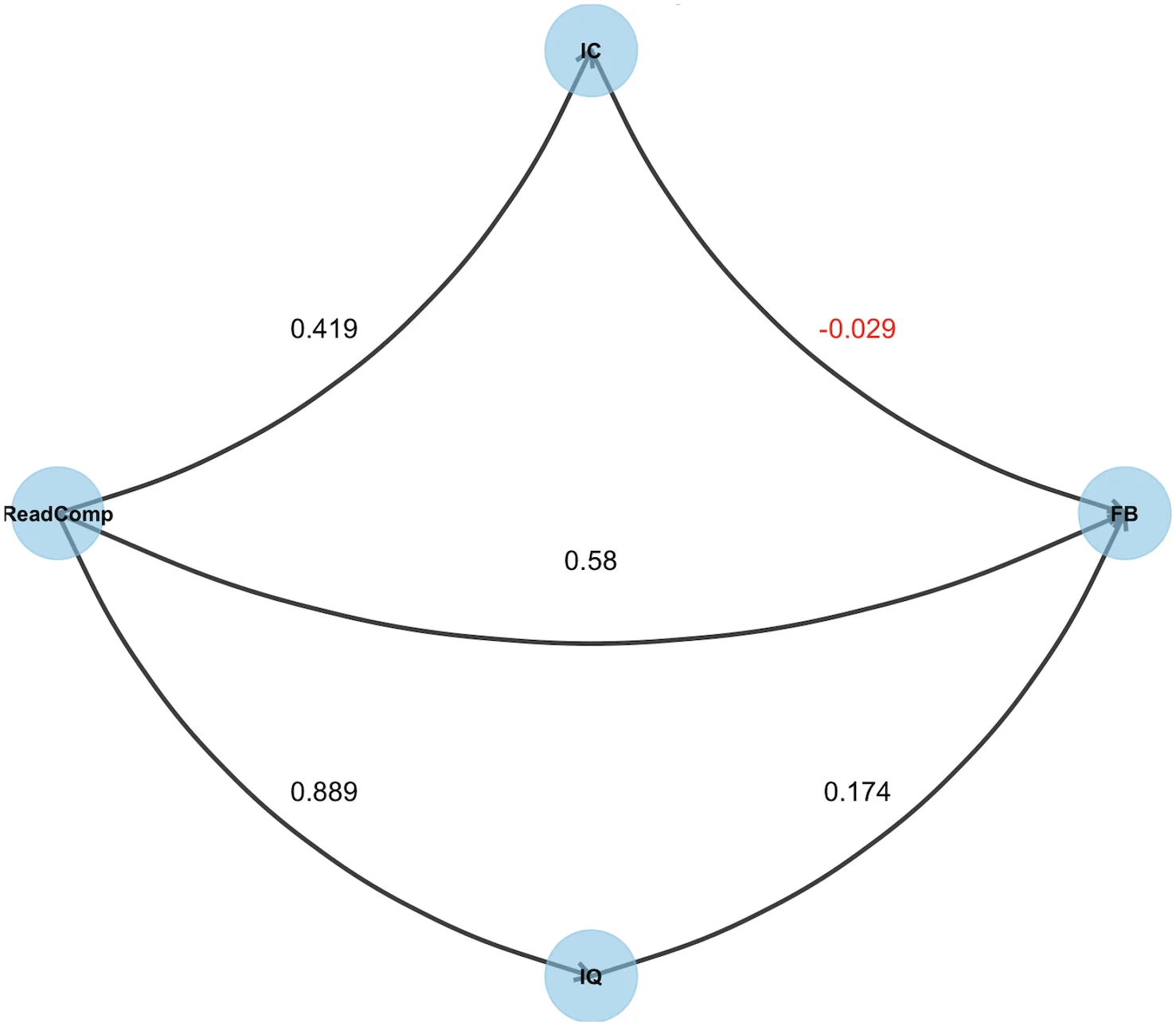
Visualization of mediation effects between reading fluency, IQ, IC and FB. *Note.* ReadComp = reading fluency.

We also explored whether the relationship between reading fluency and FB understanding is mediated by two cognitive constructs: inhibitory control (IC) and nonverbal IQ (IQ) (see Fig 6 below; see the supplementary materials for full results), using the lavaan package in R. All variables were standardized prior to analyses. The model estimated both direct and indirect effects, with indirect effects computed via bootstrapping (5,000 samples). The indirect paths were defined as: reading fluency → IC → FB and reading fluency → IQ → FB. We reported standardized coefficients and assessed model fit and explained variance (R²).

The results show that reading fluency significantly predicts both IC (β = 0.419, *p* < .001) and IQ (β = 0.889, *p* < .001), indicating strong associations with both mediators. However, neither IC (β = –0.029, *p* = .719) nor IQ (β = 0.174, *p* = .407) significantly predicts FB understanding. The direct effect of reading fluency on FB remains statistically significant (β = 0.580, *p* = .002). The indirect effects via IC (β = –0.012) and IQ (β = 0.155) were both non-significant, and the total indirect effect (β = 0.142, *p* = .447) also failed to reach significance. Overall, the total effect of reading fluency on FB understanding remains strong and significant (β = 0.722, *p* < .001), accounting for approximately 53% of the variance in FB performance.

### Comparing FB performance against previous data

**Fig 7:**
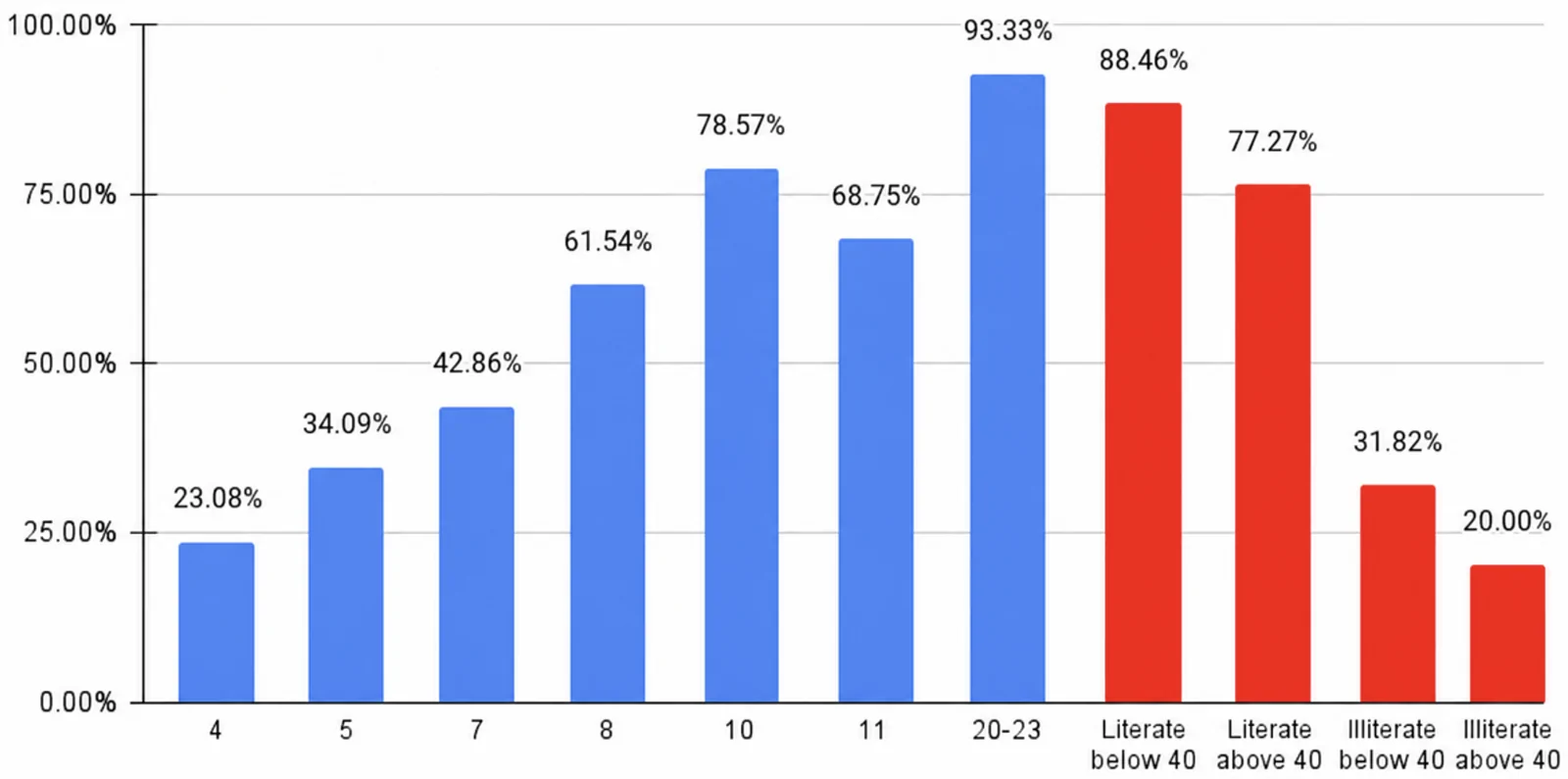
FB scores (%) obtained using the traditional scoring method from children and young adults tested by [8] (blue), and literate and illiterate speakers from this study, split into younger and older groups at age 40 (red).

In order to contextualize our results, we compare the group data from our study with data collected by [8], who administered the same FB task to Turkish speaking children of literate parents aged from kindergarten to 11 and to literate adults (see Fig 7). The data from [8] reveal a clear developmental trajectory: FB understanding improves gradually from age 4 (23%) to age 11 (68%), with a marked increase between ages 7 (42%) and 8 (61%), a shift that aligns with the beginning of formal schooling in Turkey. Peak performance is observed in the 20–23 age group (93%), suggesting that FB reasoning continues to mature throughout adolescence.

Our literate participants achieved an average score of 81%. While this is somewhat lower than the adults in the sample of [8], it is important to note that performance on false-belief tasks declines in older adults. This is clear from our regression analysis and has also been reported in earlier studies (see, for example, [79]). Thus, to facilitate meaningful comparisons, we divided both of our samples into younger (aged under 40) and older (aged above 40) subgroups. As can be seen from Fig 7, the younger literate group’s average performance is very similar to that of Ögel Balaban and Hohenberger’s adults. In stark contrast, our illiterate participants showed FB performance comparable to that of 4- to 7-year-old children, with an overall accuracy of 20%. The gap is particularly pronounced in older adults: while literate individuals over the age of 40 still performed well (77%), their illiterate peers exhibited a sharp drop (20%), highlighting the enduring effects of literacy across the lifespan.

## Discussion

To our knowledge, this is the first study to examine inhibitory control (IC) and false belief (FB) understanding in adult illiterate speakers. Our findings reveal robust and statistically significant group differences with large effect sizes across all tasks: literate participants consistently outperformed illiterate participants in FB, nonverbal IQ, and IC measures. While neither group reached ceiling-level performance on the FB tasks, literate participants came considerably closer to it. Following [10,80], if the average score of a group is below 80% of the maximum possible score, then the task can be a reliable measure of individual differences. In this vein, our FB task can detect individual differences in FB understanding in both groups reliably.

Our statistical analyses show that reading fluency emerged as the most consistent predictor of FB understanding in our models. Age also explains a small additional portion of variance, particularly in older adults, consistent with previous findings [79]. We interpret this effect of reading fluency as broadly reflecting differences associated with literacy status (i.e., literate vs. illiterate speakers): reading fluency appears to separate this literacy status more sensitively than other measures. Moreover, mediation analyses suggest that reading fluency may be a key predictor of FB understanding in our sample, though note that these analyses were exploratory and considering the relatively small sample size (N = 76) and the complexity of the multiple-mediator model, the non-significant indirect effects should not be interpreted as indicating that inhibitory control or IQ are unimportant for false belief understanding, nor that literacy eliminates the influence of these factors. However, we found no consistent support for mediation effects involving the other variables included in the dataset.

Some readers might attribute  the low performance of illiterate adults on the FB task to factors such as unfamiliarity with formal testing environments or lack of engagement. We considered these possibilities and took steps to address them. To mitigate the potential stress caused by unfamiliar settings, we established rapport with participants at least two weeks prior to data collection and tested them individually in a quiet room at their local adult education center. All tasks were administered in an informal, conversational style as games and almost all participants expressed their enjoyment after the study. Participants followed instructions attentively and performed near ceiling on the linguistic control conditions of the FB task, indicating both engagement and comprehension. That said, their lower accuracy on the reality check questions may reflect difficulty in processing visual task elements such as speech bubbles (the speech bubbles actually contained pictures rather than words but the idea is that they may not have been familiar with the convention of using speech bubbles) or specific image cues, rather than a lack of understanding of the task overall.

Another possible concern is that the stories used in the FB task may have been syntactically too difficult for adult illiterate speakers. However, this interpretation appears less likely based on our data. The stories were originally designed for children as young as four years old, and any argument that illiterates failed due to syntactic complexity would imply that illiterate individuals cannot understand simple narratives. This would be an untenable claim, given that storytelling is a feature of all cultures, including pre-literate ones [81]. Furthermore, in our study, the second-order FB question in the puppy story was grammatically simpler than the one in the chocolate story, yet illiterate participants performed better on the latter.

Sentence (2) includes subordination which sentence (1) does not, realized through the nominalizer on the verb, which is the standard subordination strategy in Turkish.

The 2nd order FB question from the puppy story:

(1) Mehmet-in anne-si anneanne-ye ne cevap ver-ir?

Mehmet-GEN mother-3SG.POSS grandmother-DAT what answer give-AOR

The 2nd order FB question from the chocolate story:

(2) Ece çikolata için Can-ın nere-ye bak-acağ-ı-nı düşün-üyor?

Ece chocolate for Can-GEN where-DAT look-FUT-NMLZ.3SG.POSS think-PRES.PROG

They also performed near ceiling on the linguistic control question in both stories. These findings suggest that syntactic difficulty and external factors did not appear to be primary determinants in the current materials. However, given the substantial contribution of reading fluency, these results should not be taken to rule out syntactic or reading-related effects in other task formats or texts, and instead may point to an interaction between FB understanding and reading fluency or reading experience. Thus, the results may differ with other FB-testing texts.

Our results showed no effect of IC or IQ on FB understanding. One possible explanation may be that prior studies demonstrating such effects have typically focused on children, where IC and IQ are still developing, and thus FB reasoning has not yet become fully developed. By contrast, our study involved a highly heterogeneous adult sample spanning the full literacy continuum, from complete illiteracy to advanced literacy. Another possibility or limitation is that we only used one task to measure IC and it is possible that a combination of multiple IC tasks may predict differences in adult FB understanding.

Be that as it may, in our regression and exploratory mediation analyses, reading fluency consistently predicted FB scores, whereas IQ and IC did not significantly contribute once reading fluency was accounted for. In light of the limited evidence for mediation and the relatively complex model structure relative to sample size, these findings should be interpreted cautiously**.** This potentially suggests that literacy, and the linguistic, cognitive, and metacognitive advantages it supports, may subsume the influence of IC and IQ in explaining FB performance in adult populations with widely varying literacy levels.

### Literacy and FB understanding

Disentangling the effects of literacy from those of education is notoriously difficult, as the two are deeply intertwined in most societies, where formal schooling typically includes systematic instruction in reading and writing (at least among hearing individuals). This overlap is evident in our dataset as well: reading fluency and years of education are highly correlated (*r* = .92, *p* < .001), making it impossible to isolate the unique contribution of either factor. Although reading fiction has been shown to improve ToM understanding to some extent (see Introduction), it is likely that both literacy and education contribute to this effect, particularly in contexts where reading fiction is embedded within formal education. Consequently, when we refer to “literacy” in this context, we mean both the ability to decode and produce written language, and the broader educational experiences that typically accompany literacy acquisition.

At the same time, we acknowledge that excluding group membership and education from the regression models due to multicollinearity constrains the interpretability of the findings. In particular, reading fluency should not be interpreted as a pure proxy for literacy alone, but rather as a composite indicator that is likely to reflect both group-related experiences and educational background. Therefore, the reported effects should be understood as reflecting shared variance among these closely intertwined factors rather than the isolated effect of any single variable.

Our analyses show that reading fluency predicts FB understanding more strongly than IQ and IC, even though the two groups differ substantially on all of these measures. This prompts a closer investigation into the role of literacy in FB understanding.

We propose several mechanisms through which learning to read and write, and practicing these skills, could influence FB performance. First, reading enriches both the quantity and quality of linguistic input (e.g., [29]), offering exposure to more complex and diverse language than oral input alone. A meta-analysis by [19] found that language abilities (i.e., vocabulary and grammar) account for 10% of the variance in FB task performance in children under seven, even after controlling for age. It is therefore plausible that differences in vocabulary and grammar knowledge in our participants contributed to group differences in FB performance.

A richer and more deeply entrenched vocabulary may support FB understanding, particularly because successful use of mental state vocabulary is crucial for passing FB tasks [82]. Literate speakers generally have larger vocabularies than illiterate speakers [83], and a large proportion of adult vocabulary is acquired through reading [84]. Supporting this view, [85] found that literate adults who read more performed better in understanding mental state verbs than those who read less. This suggests that literate speakers may perform better on FB tasks due to their greater experience with mental state vocabulary and grammar through written language.

As discussed in the Introduction, training children in subordinate clauses improves FB understanding compared to untrained children [27,28], likely because second-order FB reasoning partially depends on such syntactic structures. Literacy, in turn, offers more opportunities to acquire and consolidate complex grammatical forms (see [40,41,44,86]). Indeed, large individual differences exist in comprehending grammatical constructions such as complex noun phrases, nominalizations, subject/object relatives, and passives [40,41]. Thus, if such structures have not been reliably mastered, it may have an indirect, secondary impact on second-order FB reasoning.

Another possibility is working memory. Literacy acquisition has been shown to improve working memory capacity and there is evidence that illiterate speakers have smaller working memory capacity [42]. Considering that WM may be relevant for FB processing [68], it is plausible to assume working memory constraints might play a causal role in the FB performance of our illiterate participants. Since this study did not control for working memory, our discussion remains tentative, however future studies should consider this possibility.

Literacy may also enhance FB understanding by fostering metalinguistic awareness, i.e., the ability to reflect on and analyze the structure and function of language [87]. This includes understanding that language conveys the speaker’s intended meaning rather than directly representing reality, a skill that becomes crucial in interpreting scenarios where linguistic input misrepresents the true state of the world, as in false-belief tasks.

There is some evidence suggesting that metalinguistic awareness may support theory of mind understanding in bilingual children [88,89], although direct evidence is limited. It is therefore plausible that lower metalinguistic awareness among illiterate adults contributes to their reduced FB task performance. Without regular exposure to written language, which encourages reflection on the relationship between form and meaning, illiterate individuals may have fewer opportunities to develop metalinguistic awareness, which may impact FB understanding performance.

Reading also requires individuals to engage with the beliefs, intentions, and perspectives of others. These processes closely mirror those involved in FB understanding. In order to comprehend written narratives, readers must infer characters’ mental states, motivations, and intended meanings, relying primarily on linguistic cues rather than non-linguistic contextual information that is often available in spoken interaction. Through this process, readers are repeatedly exposed to situations that demand the interpretation and prediction of characters’ behavior based on their beliefs and desires. Such narrative exposure may serve as ongoing practice in perspective-taking and belief reasoning. Over time, this engagement with fictional minds could help strengthen the cognitive capacities underlying FB understanding, potentially supporting real-world social cognition.

A final and important consideration is that the explanations discussed above are not mutually exclusive; rather, they likely interact to produce the observed group differences. Literacy may act as a surrogate for the development of other cognitive and socio-emotional capacities. In the Turkish context, for example, women who are illiterate often come from low socioeconomic backgrounds [90], have limited access to formal education for cultural or structural reasons [91], and perform poorly on measures of IQ and grammatical comprehension in their first language [41]. In this sense, adult illiteracy can be seen as a cumulative disadvantage that affects multiple domains of cognitive and social functioning.

The following discussion provides contextual background relevant to the interpretation of group differences in FB understanding, by situating performance within the social and experiential environments associated with literacy status. Participants in our study, as well as in previous research [41,91], reported highly restricted lifestyles. Many were perceived as incompetent by their families and communities, with important personal and administrative tasks handled by male relatives. Such experiences can undermine self-confidence and autonomy [91–93], limiting social interactions beyond the immediate family and reducing exposure to cognitively demanding or socially complex situations (e.g., attending medical appointments, filing complaints, or engaging in their children’s education). These conditions may foster relatively insular, oral-based communities centered on familiar topics and roles [61,94].

This limited exposure to diverse perspectives and cognitively stimulating experiences, combined with the linguistic and cognitive mechanisms discussed above, may constrain FB understanding. This aligns with [95], who emphasize the joint role of cognitive skills and social experience in ToM development. In this context, participation in adult literacy classes and interaction with classmates, teachers, and institutional settings may provide opportunities to develop the social-cognitive capacities underlying FB understanding.

Importantly, we do not claim that illiterate individuals lack FB understanding. Testimonies provided by Yıldız [61] show that illiterate women can understand basic deception, indicating foundational ToM abilities. However, standard ToM tasks assess more advanced conceptual skills [96]. Literacy may therefore act as a symbiotic force in social cognition, both supporting and benefiting from gains in linguistic, cognitive, and ToM abilities that reinforce one another over time.

While FB understanding involves higher-level mental-state reasoning, it remains closely intertwined with language. It is therefore possible that individuals with stronger early ToM developed better language skills, attained more education, and ultimately showed stronger FB understanding. Some researchers argue that lower-level ToM skills precede and support language acquisition. For example, joint attention predicts early word learning and referential communication [97]. This is evident in autistic children without cognitive impairments, who often struggle with joint attention and, consequently, language development [98]. Early ToM also has long-term effects: ToM at age five predicts later growth in receptive vocabulary [62]. However, the illiterate participants in our study were born into agrarian families and could not attend school due to financial, cultural, or religious constraints (see [41]) and this might explain some of the differences in our results.

### Implications, future work, and limitations

While our study focuses specifically on FB understanding, it is important to recognize that ToM encompasses a broader range of abilities, including understanding desires, intentions, emotions, sarcasm, and other social-cognitive constructs. Our focus on FB, particularly second-order tasks, stems from their well-established status as benchmarks for advanced ToM reasoning. Children typically pass these tasks between the ages of 8 and 10, which coincides with formal schooling and increasing literacy.

However, because most psychological research does not report performance on these tasks in literate, typically developing adults, or restricts sampling to university students or highly literate populations, there may be an implicit assumption that FB reasoning is mastered in adulthood with minimal individual differences. Apperly [9] explicitly reflects this view, arguing there is no need to use tests such as FB to assess conceptual understanding of mental states, “because nobody really doubts that a typical adult has such basic mind reading concepts” (p. 74). Our results align with previous research showing individual differences in ToM understanding: performance on FB tasks appears more variable when assessed in individuals with limited literacy. This oversight risks masking meaningful individual differences, especially among adults with reduced access to formal education. Future research should explore whether similar patterns of disparity extend to other ToM subdomains beyond FB reasoning.

Our findings suggest that theories of ToM development should more explicitly account for the influence of literacy and the acquisition of reading and writing skills. This is in line with [62], who found that the home language and literacy environment had an indirect effect on ToM development, with language proficiency at ages 4–6 predicting later ToM performance.

Low ToM performance in adulthood can have far-reaching practical consequences. Difficulties in reasoning about others’ beliefs, intentions, or perspectives may hinder social interaction, impair relationship maintenance, and limit effective communication (e.g., [2]). Without well-developed perspective-taking skills, individuals may experience frequent misunderstandings, reduced empathy, and conflict in everyday interactions. Furthermore, challenges in inferring others’ mental states may affect problem-solving in social contexts, such as resolving disputes or negotiating shared goals.

A key domain where ToM skills are especially critical is referential communication, where speakers must assess the listener’s knowledge state and tailor their message accordingly [99]. This ability is crucial for successful communication, particularly in high-stakes interactions with teachers, medical professionals, or law enforcement officials [100]. Individuals with low ToM performance may struggle in such contexts, leading to frequent misunderstandings or communication breakdowns [101]. Recent evidence suggests that this may be especially pronounced among illiterate adults [102], further highlighting the practical consequences of limited ToM reasoning in adult populations.

This study has several limitations that should be taken into account. First, the sample size was relatively small, limiting the generalizability of the findings. Future research should aim to include larger and more diverse illiterate populations from different regions to ensure broader applicability. Indeed, these findings may not generalize to men since we could only collect data from illiterate women. Second, the study relied on a limited set of ToM tasks. Incorporating a wider range of tasks would provide a more nuanced picture of how literacy affects different facets of ToM. Third, the correlational nature of the data prevents strong causal claims; future studies using longitudinal or experimental designs would help clarify the direction of effects. Fourth, we were unable to test working memory because of limited time. Since working memory likely plays a significant role in second-order false belief tasks, future researchers should include this measure, especially when studying illiterate speakers, to gain a deeper understanding of the results. Finally, future research may wish to use a paragraph based reading fluency measure rather than the task used here.

Additional factors warrant further investigation. For example, measures of reading comprehension and reading habits could help disentangle the effects of literacy experience more precisely. Longitudinal studies could also shed light on how the relationship between literacy and ToM unfolds across the lifespan, including during childhood. Moreover, exploring the role of language skills, such as mental-state vocabulary and syntactic complexity (e.g., subordination), may help determine whether the observed effects are mediated by broader linguistic abilities. Understanding these pathways would allow researchers to better isolate the unique contribution of literacy to ToM development.

## Conclusion

This study offers novel insights into the relationship between literacy and false-belief (FB) understanding in illiterate and literate adults. Our findings suggest that acquiring literacy, and the broader cognitive and linguistic changes it entails, play a crucial role in enhancing FB reasoning. A possible interpretation of these results is that literacy acquisition may support FB reasoning through increased exposure to enriched linguistic input via written language, improved working memory, heightened metalinguistic awareness, and the cognitive demands of interpreting written texts, which may promote perspective-taking. Reading ability emerged as the strongest predictor of FB task performance, explaining 49% of the variance. This highlights the role of literacy as a foundational factor supporting both cognitive and linguistic development, which in turn facilitates FB understanding.

These findings have broad implications for both psychology and adult education. By studying a previously untapped population, this research challenges established cognitive norms and provides a different insight to perspective-taking. In psychology, they emphasize the importance of literacy in social-cognitive processes such as perspective-taking. In adult education, the results advocate for literacy programs not only as tools for communication but also as means to enhance cognitive and social skills. Overall, this study reinforces the significant impact of literacy on cognitive development and calls for educational strategies that support reading acquisition, especially for individuals with limited access to formal education.

## Supporting information

S1 Table Correlations between variables in the literate group. (DOCX)

S2 Table Correlations between variables in the illiterate group. (DOCX)

S3 Table Correlations between variables in the full sample. (DOCX)

S4 Table Initial full model (predicting sensitive FB). (DOCX)

S5 Table Final best fitting model (predicting sensitive FB) when reading fluency is swapped with education. (DOCX)

S2 File False-belief images used during story narration. (DOCX)

S3 File Inclusivity in global research. (DOCX)

S4 File Frequency analysis of content words in the two Theory of Mind tasks. (DOCX)

S5 File Link to the false-belief stories. (DOCX)
