# Supplementary material for: Literacy predicts performance on a second-order false-belief task
Source: PLoS One. 2026 Sep 2;21(9):e0357313. doi: 10.1371/journal.pone.0357313 (PMC13537559; doi:10.1371/journal.pone.0357313)
Supplement: Supplementary file 1 [file pone.0357313.s001.docx]

CORRELATIONS NOT BONFERRONI CORRECTED OR NOT CORRECTED FOR MULTIPLE COMPARISONS

**S1 Table 1a. Correlations between variables in the literate group.**

| Var | Age | Years Edu | Read Real | Read Pseudo | IC | IQ | FB Sensitive | FB Traditional | Reading fluency |
| --- | --- | --- | --- | --- | --- | --- | --- | --- | --- |
| Age | — | -0.49** | -0.38* | -0.24 | 0.04 | -0.33 | -0.39* | -0.38* | -0.34* |
| Years Edu | -0.49** | — | 0.29 | 0.40* | 0.28 | 0.47** | 0.25 | 0.20 | 0.41* |
| Read Real | -0.38* | 0.29 | — | 0.50** | -0.09 | 0.17 | 0.05 | 0.27 | 0.81*** |
| Read Pseudo | -0.24 | 0.40* | 0.50** | — | -0.15 | 0.34* | 0.09 | 0.09 | 0.91*** |
| IC | 0.04 | 0.28 | -0.09 | -0.15 | — | 0.01 | 0.00 | -0.04 | -0.14 |
| IQ | -0.33 | 0.47** | 0.17 | 0.34* | 0.01 | — | 0.16 | 0.07 | 0.31 |
| FB Sensitive | -0.39* | 0.25 | 0.05 | 0.09 | 0.00 | 0.16 | — | 0.77*** | 0.08 |
| FB Traditional | -0.38* | 0.20 | 0.27 | 0.09 | -0.04 | 0.07 | 0.77*** | — | 0.19 |
| Reading fluency | -0.34* | 0.41* | 0.81*** | 0.91*** | -0.14 | 0.31 | 0.08 | 0.19 | — |

*Note.* *p* < .05, **p** < .01, ***p*** < .001. IC: Inhibitory control, Years Edu: years spent in formal education
