# Supplementary material for: Literacy predicts performance on a second-order false-belief task
Source: PLoS One. 2026 Sep 2;21(9):e0357313. doi: 10.1371/journal.pone.0357313 (PMC13537559; doi:10.1371/journal.pone.0357313)
Supplement: Supplementary file 2 [file pone.0357313.s002.docx]

CORRELATIONS NOT BONFERRONI CORRECTED OR NOT CORRECTED FOR MULTIPLE COMPARISONS

**S1 Table 1b. Correlations between variables in the illiterate group.**

|  | Age | Years Edu | Read Real | Read Pseudo | IC | IQ | FB Sensitive | FB Traditional | Reading fluency |
| --- | --- | --- | --- | --- | --- | --- | --- | --- | --- |
| Age | — | -0.20 | 0.10 | 0.00 | -0.03 | -0.27 | -0.20 | -0.15 | 0.07 |
| Years Edu | -0.20 | — | -0.06 | -0.09 | 0.11 | 0.33* | 0.07 | 0.05 | -0.07 |
| Read Real | 0.10 | -0.06 | — | 0.94*** | -0.13 | 0.28 | 0.30 | 0.18 | 0.99*** |
| Read Pseudo | 0.00 | -0.09 | 0.94*** | — | -0.16 | 0.21 | 0.34* | 0.23 | 0.97*** |
| IC | -0.03 | 0.11 | -0.13 | -0.16 | — | 0.26 | 0.03 | 0.02 | -0.14 |
| IQ | -0.27 | 0.33* | 0.28 | 0.21 | 0.26 | — | 0.09 | 0.04 | 0.27 |
| FB Sensitive | -0.20 | 0.07 | 0.30 | 0.34* | 0.03 | 0.09 | — | 0.85*** | 0.32* |
| FB Traditional | -0.15 | 0.05 | 0.18 | 0.23 | 0.02 | 0.04 | 0.85*** | — | 0.20 |
| Reading fluency | 0.07 | -0.07 | 0.99*** | 0.97*** | -0.14 | 0.27 | 0.32* | 0.20 | — |

*Note.* *p* < .05, **p** < .01, ***p*** < .001. IC: Inhibitory control, Years Edu: years spent in formal education
