# Supplementary material for: Literacy predicts performance on a second-order false-belief task
Source: PLoS One. 2026 Sep 2;21(9):e0357313. doi: 10.1371/journal.pone.0357313 (PMC13537559; doi:10.1371/journal.pone.0357313)
Supplement: Supplementary file 3 [file pone.0357313.s003.docx]

CORRELATIONS NOT BONFERRONI CORRECTED OR NOT CORRECTED FOR MULTIPLE COMPARISONS

**S1 Table 1c. Correlations between variables in the full sample.**

| Var | Age | Years Edu | Read Real | Read Pseudo | IC | IQ | FB Sensitive | FB Traditional | Reading fluency |
| --- | --- | --- | --- | --- | --- | --- | --- | --- | --- |
| Age | — | -0.26* | -0.18 | -0.19 | -0.07 | -0.26* | -0.29* | -0.27* | -0.19 |
| Years Edu | -0.26* | — | 0.90*** | 0.92*** | 0.51*** | 0.90*** | 0.76*** | 0.71*** | 0.92*** |
| Read Real | -0.18 | 0.90*** | — | 0.94*** | 0.43*** | 0.88*** | 0.76*** | 0.73*** | 0.99*** |
| Read Pseudo | -0.19 | 0.92*** | 0.94*** | — | 0.43*** | 0.88*** | 0.75*** | 0.70*** | 0.99*** |
| IC | -0.07 | 0.51*** | 0.43*** | 0.43*** | — | 0.51*** | 0.39*** | 0.36*** | 0.43*** |
| IQ | -0.26* | 0.90*** | 0.88*** | 0.88*** | 0.51*** | — | 0.73*** | 0.66*** | 0.89*** |
| FB Sensitive | -0.29* | 0.76*** | 0.76*** | 0.75*** | 0.39*** | 0.73*** | — | 0.91*** | 0.77*** |
| FB Traditional | -0.27* | 0.71*** | 0.73*** | 0.70*** | 0.36*** | 0.66*** | 0.91*** | — | 0.72*** |
| Reading fluency | -0.19 | 0.92*** | 0.99*** | 0.99*** | 0.43*** | 0.89*** | 0.77*** | 0.72*** | — |

*Note.* *p* < .05, **p** < .01, ***p*** < .001. IC: Inhibitory control, Years Edu: years spent in formal education
