# Supplementary material for: Literacy predicts performance on a second-order false-belief task
Source: PLoS One. 2026 Sep 2;21(9):e0357313. doi: 10.1371/journal.pone.0357313 (PMC13537559; doi:10.1371/journal.pone.0357313)
Supplement: Supplementary file 4 [file pone.0357313.s004.docx]

**S1 Table 1d. Initial full model (predicting sensitive FB).**

| **Predictor Variable** | **Estimate** | **Standard error** | ***t* value** | **Pr(> \|t\|)** |
| --- | --- | --- | --- | --- |
| **Intercept** | 0.000 | 0.073 | 0.00 | 1 |
| **Inhibitory control** | 0.056 | 0.085 | 0.650 | 0.518 |
| **IQ** | 0.107 | 0.172 | 0.619 | 0.538 |
| **Age** | -0.144 | 0.076 | -1.882 | 0.064 |
| **Reading fluency** | 0.623 | 0.161 | 3.866 | < .001 |
| **R²= .5977** |  |  |  |  |
