# Supplementary material for: Literacy predicts performance on a second-order false-belief task
Source: PLoS One. 2026 Sep 2;21(9):e0357313. doi: 10.1371/journal.pone.0357313 (PMC13537559; doi:10.1371/journal.pone.0357313)
Supplement: Supplementary file 5 [file pone.0357313.s005.docx]

**S1 Table 1e. Final best fitting model (predicting sensitive FB) when reading fluency is swapped with education.**

| **Predictor Variable** | **Estimate** | **Standard error** | ***t* value** | **Pr(> \|t\|)** |
| --- | --- | --- | --- | --- |
| **Intercept** | 0.00 | 0.07 | 0.00 | 1.00 |
| **Education** | 0.68 | 0.08 | 8.17 | .000 |
| **R²=** .4747 |  |  |  |  |
