# Supplementary material for: Literacy predicts performance on a second-order false-belief task
Source: PLoS One. 2026 Sep 2;21(9):e0357313. doi: 10.1371/journal.pone.0357313 (PMC13537559; doi:10.1371/journal.pone.0357313)
Supplement: Supplementary file 6 [file pone.0357313.s006.docx]

Images used in this study were originally drawn by Flobbe, L. (2006). Children's development of reasoning about other people's minds (Doctoral dissertation, Faculty of Science and Engineering, University of Groningen).

Please follow the link to find the AI enhanced images used in our study (file name: False-Belief Booklet.pptx): <https://osf.io/9mhgv/?view_only=43b29ab2fc6940a89f7f403ab69303e2>

See also the same images attached to this document as well.

Puppy story
