# Supplementary material for: Literacy predicts performance on a second-order false-belief task
Source: PLoS One. 2026 Sep 2;21(9):e0357313. doi: 10.1371/journal.pone.0357313 (PMC13537559; doi:10.1371/journal.pone.0357313)
Supplement: Supplementary file 8 [file pone.0357313.s008.docx]

To verify that the story materials were linguistically appropriate for the participant population, we examined the lexical frequency of all content words appearing in the stories. Given that the stories were originally designed for use with children as young as four years of age, they were expected to contain highly familiar, everyday vocabulary. Nevertheless, we assessed this assumption empirically by consulting the Turkish data available in the CHILDES database (<https://talkbank.org/childes/access/Other/Turkish/Aksu.html>), using speech produced by children between 2 years and 4 years 4 months of age. The vast majority of lexical items occurred at least five times in this corpus, indicating that they are common in early child-directed and child-produced language.

For the 13 lexical items that occurred fewer than five times in CHILDES, we additionally examined their normalized frequencies (occurrences per million words) in the spoken subcorpus of the Turkish National Corpus (TNC). As shown in Table 1 below, nearly all of these words were also relatively frequent in spoken Turkish. The only exceptions were the onomatopoeic expression *zır zır* (“ring ring”) and the compound *meyve kabukları* (“fruit peels”), which did not occur in the spoken TNC subcorpus, although both are attested in the written subcorpus. Overall, these analyses indicate that the stories consisted almost entirely of high-frequency vocabulary that would be expected to be familiar to adult native speakers regardless of literacy level.

**Table 1.** **Normalized frequencies of lexical items with fewer than five occurrences in the CHILDES Turkish corpus, based on the spoken subcorpus of the Turkish National Corpus (TNC)**

| Turkish word | English translation | Per million frequency in the spoken subcorpus of the TNC |
| --- | --- | --- |
| 1- Sürpriz | Surprise | 36.49 |
| 2- Paten | Roller skate | 31.56 |
| 3- Zır zır | Ring ring (onomatopoeia) | 0 (though 0.95 in the written subcorpus) |
| 4- Parti | Party | 725.82 |
| 5- Gerçekten | Really | 761.32 |
| 6- alışveriş | Shopping | 47.34 |
| 7- Çikolata | chocolate | 41.42 |
| 8- Kalan | Remaining | 929.96 |
| 9- Sinirlenmek | To get angry | 75.94 |
| 10- Meşgul | Busy | 29.59 |
| 11- Meyve kabukları | Fruit peels | 0 (though 14.59 in the written subcorpus) |
| 12- sandık | Chest | 104.53 |
| 13- düşünmek | To think | 1617 |
