# Supplementary material for: Literacy predicts performance on a second-order false-belief task
Source: PLoS One. 2026 Sep 2;21(9):e0357313. doi: 10.1371/journal.pone.0357313 (PMC13537559; doi:10.1371/journal.pone.0357313)
Supplement: Supplementary file 9 [file pone.0357313.s009.docx]

<https://osf.io/9mhgv/files/osfstorage/682366899211f5be57eae0fa>
